# Multi-Year Monitoring of Deciduous Forests Ecophysiology and the Role of Temperature and Precipitation as Controlling Factors

**DOI:** 10.3390/plants11172257

**Published:** 2022-08-30

**Authors:** Stavros Stagakis, Nikos Markos, Theofilos Vanikiotis, Efi Levizou, Aris Kyparissis

**Affiliations:** 1 Department of Environmental Sciences, University of Basel, Klingelbergstrasse 27, 4056 Basel, Switzerland; 2 Department of Biological Applications & Technology, University of Ioannina, Leof. S. Niarchou, 451 10 Ioannina, Greece; 3 Department of Agriculture Crop Production and Rural Environment, School of Agricultural Sciences, University of Thessaly, Fytokou Str., N. Ionia, 384 46 Volos, Greece

**Keywords:** *Fagus sylvatica*, *Quercus cerris*, *Quercus frainetto*, ecophysiology, climatic control, photosynthesis, water potential, LAI, chlorophylls, Mediterranean

## Abstract

Two deciduous forest ecosystems, one dominated by *Fagus sylvatica* and a mixed one with *Quercus cerris* and *Quercus frainetto*, were monitored from an ecophysiological perspective during a five-year period, in order to assess seasonal fluctuations, establish links between phenology and ecophysiology, and reveal climatic controls. Field measurements of leaf area index (LAI), chlorophyll content, leaf specific mass (LSM), water potential (Ψ) and leaf photosynthesis (Aleaf) were performed approximately on a monthly basis. LAI, chlorophylls and LSM fluctuations followed a recurrent pattern yearly, with increasing values during spring leaf burst and expansion, relatively stable values during summer and decreasing values during autumn senescence. However, pre-senescence leaf fall and chlorophyll reductions were evident in the driest year. The dynamically responsive Aleaf and Ψ presented considerable inter-annual variation. Both oak species showed more pronounced depressions of Aleaf and Ψ compared to beech, yet the time-point of their appearance coincided and was the same for all species each year. Spring temperature had a positive role in the increasing phase of all ecophysiological processes while rising autumn temperature resulted in retarded senescence. Precipitation showed asymmetric effects on the measured ecophysiological parameters. The between-species differences in responses, climate sensitivity and climate memory are identified and discussed.

## 1. Introduction

A tree’s function is the apparent outcome of the interplay between ecophysiology, phenology, morpho-anatomical development and climate. These interactions are characteristicallypronounced on deciduous trees due to the intense seasonal fluctuations of the above-mentioned processes. Bud burst during spring, for instance, is a major phenological stage, which depends on carbohydrate storage (ecophysiology of the previous season), winter temperature, precipitation and photoperiod (environment), as well as the concurrent cambial activity and radial growth (plant development). In turn, the timing, speed and extent of bud and leaf burst determine photosynthetic performance, thus the stream of new photoassimilates for supporting foliage development (ecophysiology), the rate of which is largely mediated by temperature and precipitation. Accordingly, an integrated approach of both phenological and ecophysiological attributes of deciduous trees in relation to climatic controlling factors is needed to describe seasonal fluctuations in tree function and project this information to future conditions in the frame of the climate crisis. Moreover, the experimental framework of this integrated approach is also very important when working with deciduous trees. Time-scale, tree age and the mode of the environmental pressure imposition critically affect plant responses [1,2,3]. In fact, short-growth experiments with tree samplings and acute stresses typically monitor short-term plant responses since plants can only be partially acclimated to the prevailing conditions [4]. On the contrary, field monitoring of mature forest stands directly reflects mainly long-term acclimation processes, based on genetic and physiological traits of trees living for a long time under specific conditions. Of course, in the latter case, short-term responses to fluctuating meteorological conditions are also recorded, depicting the mature tree‘s tolerance and resilience or sensitivity.

*Fagus sylvatica* is a tree species that shows a considerable plasticity in its responses to different environments as judged by its wide distribution in various latitudes, altitudinal zones, soil chemical and water conditions [4,5,6]. Nevertheless, it is considered relatively drought-sensitive, although differences between genotypes are evident; genotypes originating from its southern distribution limit show greater tolerance in comparison with central European ones, further highlighting species plasticity [7,8,9,10]. Metabolic and genetic adaptations of beech to the particularly harsh Mediterranean environment have been identified that account for the observed tolerance, such as the increase in water use efficiency in low-altitude beech populations in Spain [11], adjusted nitrogen, carbon and water balance under extreme drought in Greece [12], and moreover temperature-related genetic alterations of individuals during the last half of 20th century in parallel with temperature rise in Spain [13]. However, recent reports have highlighted that beech populations are most likely to be endangered by temperature rise, suffering growth decline, dieback and displacement in Italy and Spain, i.e., at their southern distribution edge [6,7]. Concerns have been also expressed for species’ growth and vitality in drought-affected regions in the core of its distribution [10], given the fact that severe drought events of the last twenty years doubled or tripled beech mortality in these areas [2,14].

*Quercus cerris* and *Q. frainetto* usually occupy more xeric areas with lower elevation compared to *F. sylvatica*. They are therefore more exposed to drought conditions, facing them with a suite of traits, which correspond to a drought tolerance strategy, if the water shortage is not too prolonged [15,16]. The reduced susceptibility of oaks to water stress compared to beech has been ascribed to their slower growth rates, along with the different radial growth phenology including early season wood formation in oaks compared to beech [10,17]. The among-species differences in the perception of and response to temperature and water stress may have serious implications in light of climate warming. Kasper et al. [10] suggested that the replacement of beech by the more drought-tolerant oak species in the dry end (elevational or latitudinal) of beech distribution is likely in the near future, corroborating earlier reports [18,19].

Beech and oak forests in mixed or mono-specific stands are the most extensive broadleaf forests in Greece [20], the majority of which fall within the limits of protected National Parks. *Q. cerris* and *Q. frainetto* have been rarely studied [21,22], being two rather neglected oak species, possibly due to the dominance of other congeneric species in the European forests. On the other hand, the phenology of beech has been extensively studied in central European populations, with emphasis on spring budburst and much less attention to autumn senescence events [23,24]. Nonetheless, in southeastern distribution limits, there is a void of information about phenology and the related climate impact. Ecophysiological measurements in naturally occurring forests are also scarce in both central and south European sites and are usually restricted to 3–4 time points during the green tree phase. Finally, the detailed mapping of climatic control on the most representative phenological events and ecophysiological processes is urgently needed to assess the crucial factors that shape trees’ performance, and affect the species-specific carbon- and water-use responses and strategies, which may influence their competitive relationships. The aim of the present study was two-fold as follows: a) to perform a detailed and long-term monitoring of phenological and ecophysiological responses of a naturally occurring mono-specific beech forest and a mixed forest of *Q. cerris* and *Q. frainetto*, under field conditions during five years, and b) to examine these trees’ responses against temperature and precipitation data to reveal the climatic control on beech and oaks at their southeastern distribution limit.

## 2. Materials and Methods

### 2.1. Study Sites

**Figure 1:**
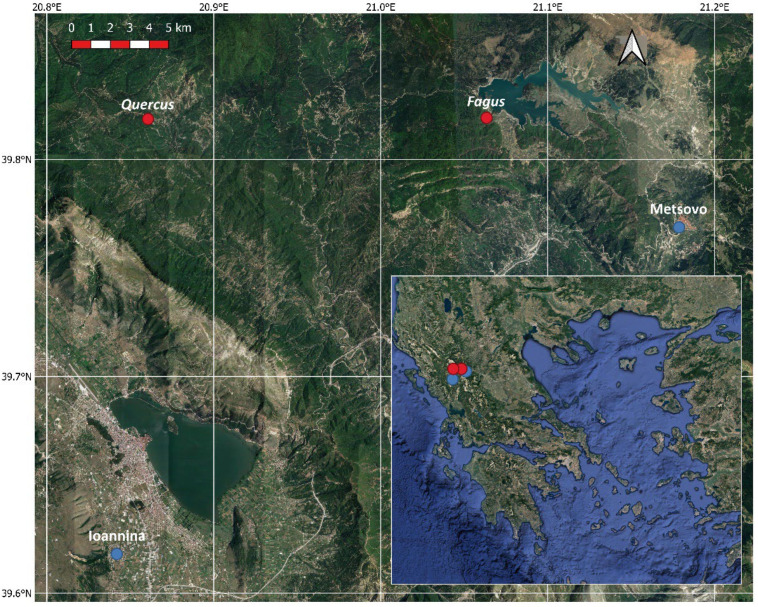
Google Satellite image of the study area, Greece (inset), with the studied sites indicated with red dots and the meteorological stations with blue dots.

The study sites are located in North Pindus National Park (area 1970 km^2^) in northwestern Greece (Lat 39.97° N, Lon 21.01° E). The National Park includes 15 different forest habitats, with most of the forest area (1041 km^2^) covered by black pine (*Pinus nigra*), deciduous oaks (*Quercus frainetto*, *Quercus cerris*), beech (*Fagus sylvatica*), fir (*Abies borisii-regis*) and Bosnian pine (*Pinus heldreichii*). The elevation of the park ranges from 400 to 2637 m and the climate is variable according to altitude and topography, ranging from mesomediterranean to submediterranean [25]. Two study sites were selected for this study, one dominated by *Fagus sylvatica* and a mixed one with *Quercus frainetto* and *Quercus cerris* in similar abundances (Figure 1). Both study sites are natural mature forests with dense canopies of approximate height between 25 and 30 m, extending for at least 25 km^2^ and containing a mixture of young and mature trees. From both forests, we selected a representative stand of 1 km^2^ with mature and uniform trees to perform our study.

### 2.2. Field Measurements

Two sets of field measurements were conducted during a three-year period (2005 to 2007) and a two-year period (2013 to 2014). Measurements were performed approximately once per month for each species, except for the spring period when more frequent measurements were made to capture the rapid developmental processes.

Leaf area index (LAI, m^2^ m^−2^) was measured using an AccuPAR LP-80 PAR/LAI Ceptometer (Decagon Devices, Inc., Pullman, Washington, DC, USA) following Norman and Jarvis model [26] of radiation transmission and scattering. LAI measurements covered two parallel to the slope 100 m transects of East-West orientation at each study site.

Photosynthesis (Aleaf, μmol CO_2_ m^−2^ s^−1^) measurements were performed with a portable photosynthesis system (LCpro+, ADC BioScientific Ltd., Hoddesdon, UK). Instantaneous photosynthesis of about 25 randomly selected leaves of the outer canopy from 8 to 10 mature trees (2–3 leaves per tree) was measured during midday at ambient conditions.

Midday leaf water potential (Ψ, MPa) was measured using a Scholander-type pressure chamber (SKPM 1400, Skye Instruments Ltd., Llandrindod Wells, UK). About 8 to 10 samples from the outer part of the canopy from several mature trees (1 sample per tree) randomly distributed along each site were wrapped in aluminum foil, sealed in plastic bags for 10 min and then cut and measured immediately with the pressure chamber.

Photosynthetic pigments and leaf specific mass (LSM) measurements were performed on the same leaves with photosynthesis measurements. After gas-exchange measurements, leaves were cut, sealed in air-tight plastic bags, transferred to the laboratory and used immediately. Chlorophylls were extracted with 80% *v*/*v* acetone and estimated spectrophotometrically using a Hitachi U-2800 double beam UV-VIS spectrophotometer (Tokyo, Japan), according to [27]. LSM was calculated as dry mass per leaf area, after drying in the oven at 80 °C for 24 h.

### 2.3. Meteorological Data

**Figure 2:**
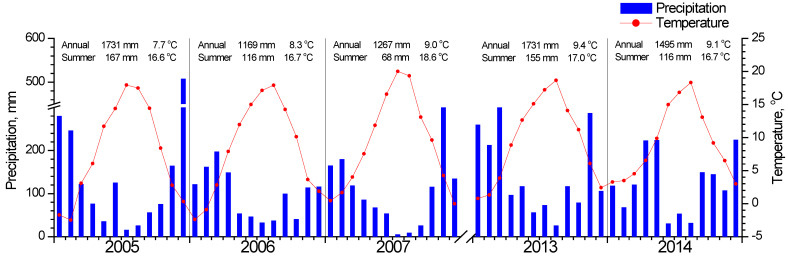
Meteorological data for the studied years. Average monthly temperature is indicated with the red circles and total monthly precipitation with blue bars. The annual and summer (June, July, August) total precipitation and average temperature are shown at the top of each year graph.

Meteorological data (average daily temperature and daily precipitation) for the study period (Figure 2) were recorded by a meteorological station situated in Metsovo (Epirus, Greece), about 11 and 27 km from the *F. sylvatica* and *Quercus* study sites respectively (Figure 1). Missing data were filled by using data from Ioannina meteorological station (Epirus, Greece, 33 km from Metsovo) after linear regressions with Metsovo data. Data for both stations were downloaded from the National Observatory of Athens (https://meteo.gr/, accessed on 1 March 2022).

### 2.4. Statistics

The relationships between field-measured parameters and climatic variables were examined through correlation analyses, performed with the JASP software v.0.16 (JASP Team 2021 Computer Software, Amsterdam, The Netherlands).

Since the examined plant parameters show rapid changes during spring and autumn and approximately stable values during summer–except for water potential and photosynthesis, which appear different patterns (see Results section)–the dataset was split into three parts for spring (measurements up to June 15), summer (measurements between June 15 and September 15) and autumn (measurements after September 15). Accordingly, for each parameter per species and season the correlations with temperature and precipitation were examined for several 5-day intervals and lags up to 90 days before measurements. Correlation analysis was performed through single linear regressions (Pearson correlations) between plant parameters and climatic variables.

## 3. Results

### 3.1. Leaf Area Index

**Figure 3:**
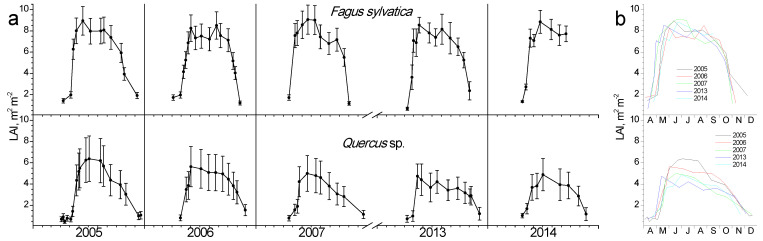
Seasonal fluctuation of leaf area index (LAI) for the two study sites. (**a**) Data for each year separately (average values ± SD for each date) (**b**) data for all years (only average values per date, error bars are not shown for clarity reasons).

In Figure 3, the seasonal fluctuation of LAI for the two study sites is shown. One LAI was measured for the two *Quercus* species since they form a mixed stand with approximately similar abundance. All species that burst their new leaves from mid-April to early May and expand them rapidly until the end of May. This spring phenological pattern was repeated every spring; however, the exact dates of leaf burst and expansion showed shifts (10–25 days) from year to year (Figure 3b). Beech seems to complete leaf expansion more rapidly compared to the two *Quercus* species. During summer, LAI is kept at high values in the beginning and gradually decreases until the autumn onset when it shows a rapid decrease, corresponding to leaf fall. The year 2007 was characterized by an exceptionally dry summer, with total precipitation during the June-August period being only 68 mm, almost half of the other studied years’ average (Figure 2). In 2007, a mid-summer LAI decrease was observed in all species, corresponding to a pre-senescence leaf fall. In all years, beech tended to sustain LAI values near the maximum for longer periods during summer and leaf fall occurred much more rapidly compared to *Quercus*. Additionally, its maximum (around 8 m^2^ m^−2^) was higher than *Quercus* (5 m^2^ m^−2^).

### 3.2. Chlorophylls

**Figure 4:**
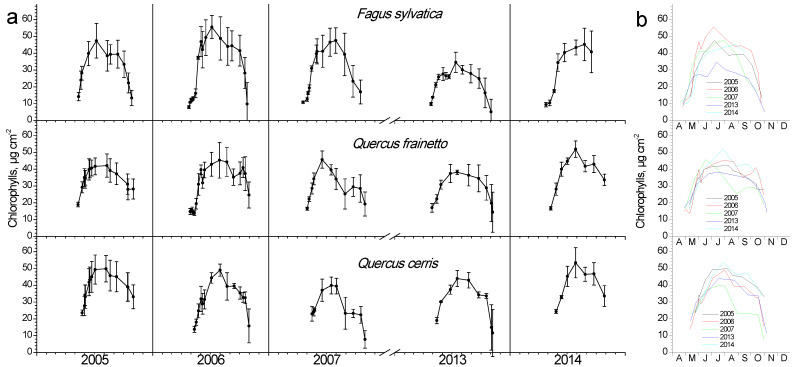
Seasonal fluctuation of leaf chlorophyll content for the three studied species. (**a**) Data for each year separately (average values ± SD for each date) (**b**) data for all years (only average values per date, error bars are not shown for clarity reasons).

Chlorophyll content showed an intra-annual pattern similar to LAI (Figure 4). As leaves burst and expand during April and May, they also increase their pigment content, keeping them at high values during summer and gradually decrease them during autumn. In beech, pigment composition during spring occurred more rapidly compared to the two *Quercus* species. Inter-annual differentiations during spring were not as apparent as in LAI; however, large differences during mid-summer maxima were evident for all three species (Figure 4b). During the dry year of 2007, a considerable decline in chlorophyll content for both *Quercus* species, but interestingly not for beech, was observed, unlike the LAI response of the latter during that year. The average chlorophyll content was similar among species.

### 3.3. Leaf Specific Mass

**Figure 5:**
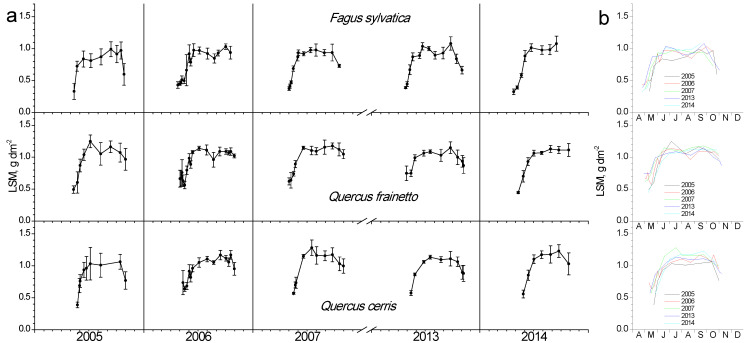
Seasonal fluctuation of leaf specific mass (LSM) for the three studied species. (**a**) Data for each year separately (average values ± SD for each date) (**b**) data for all years (only average values per date, error bars are not shown for clarity reasons).

Similar to LAI and chlorophyll content, leaf specific mass (LSM) showed a rapid increase during spring and kept stable values during summer, while only small decreases were evident during autumn (Figure 5). Additionally, only small differentiations between species and years have been observed. The only noteworthy is a trend for increased values in *Q. cerris* in the dry 2007.

### 3.4. Water Potential

**Figure 6:**
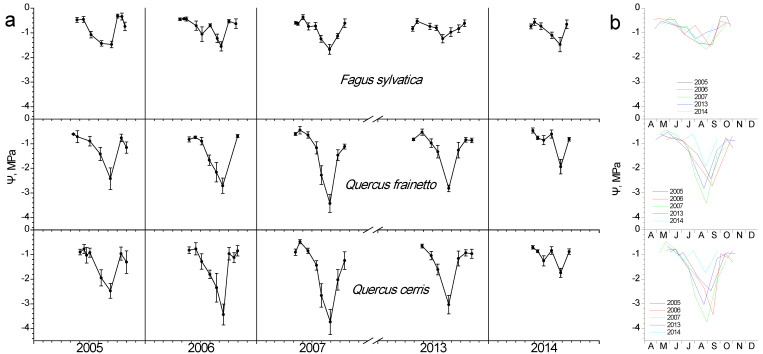
Seasonal fluctuation of leaf water potential (Ψ) for the three studied species. (**a**) Data for each year separately (average values ± SD for each date) (**b**) data for all years (only average values per date, error bars are not shown for clarity reasons).

Regardless of species, water potential showed high values during spring–early summer, when water is still ample after the winter rainy period (Figure 6). As the summer dry period advances, depressions in water potential appear for all species. A gradual decline was evidently reaching a minimum Ψ, the time-point of which was similar in all species each year, despite their differences in the exact value. A year-to-year variation in the date of minimum Ψ is obvious in Figure 6b. The two *Quercus* species seem to suffer stronger water stress compared to beech, showing lower Ψ during all study years, with 2007 being the most stressful one. The amplitude of Ψ decline is higher in *Q. cerris*, which in 2007, for instance, dropped from −0.46 MPa in late May to −3.7 MPa in late August, with the corresponding values being −0.5 to −3.4 MPa for *Q. frainetto* and −0.4 to −1.67 MPa for beech. After the onset of autumn rains, water potential recovered to high values for all species.

### 3.5. Photosynthesis

**Figure 7:**
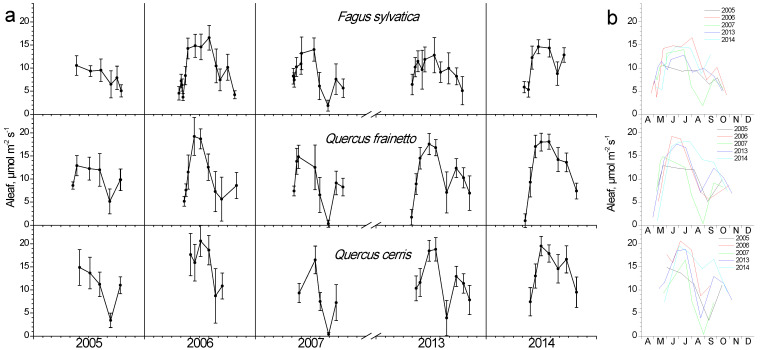
Seasonal fluctuation of leaf photosynthesis (Aleaf) for the three studied species. (**a**) Data for each year separately (average values ± SD for each date) (**b**) data for all years (only average values per date, error bars are not shown for clarity reasons).

During the leaf-out phase (spring), when the photosynthetic apparatus is not fully developed and the prevailing temperature is still low, photosynthesis shows minimum values yet gradually increases (Figure 7). During summer, the combination of high temperature and water shortage resulted in strong depressions in photosynthesis for all species, especially during years with strong water potential depressions (compare with Figure 6). Even though the *Quercus* species seem to be more affected, a strong depression was also apparent for beech during 2007, i.e., the year with the lowest water potential values. The time-point of the summer minimum in photosynthesis coincided with the minimum measured Ψ in all years. Beech retained high photosynthetic rates for longer periods throughout the green phase, while in oaks the peak values started declining during mid-July in 3 out of 5 years. Nevertheless, *Q. cerris* presented both the highest and the lowest absolute Aleaf values in all years compared to the other two species, without considerable differences with *Q. frainetto*. All species recovered their photosynthetic performance to some extent during autumn, followed by a gradual decrease during leaf senescence.

### 3.6. Climatic Control

Τhe above-described parameters were examined against temperature and precipitation, the basic climatic factors, which are expected to influence plant phenology and physiology in Mediterranean ecosystems. As described in Materials and Methods, the dataset was split into three parts for spring, summer and autumn and each plant parameter per species and season was examined against temperature and precipitation for several 5-day intervals and lags up to 90 days before measurements.

**Figure 8:**
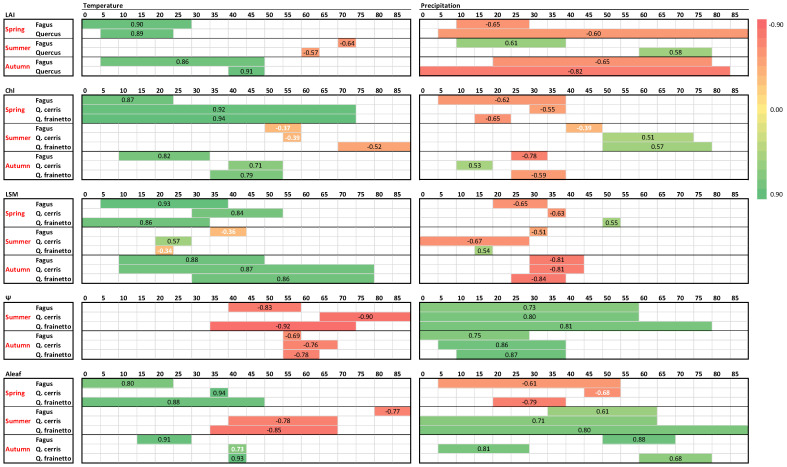
Climatic control on ecophysiological parameters of the three studied species as derived by single linear regressions. For each parameter per species and season, the regression coefficients (R) of the most significant relationships with temperature and precipitation and the corresponding time period are presented in the colored horizontal lines, according to the chromatic scale appearing in the right. Each small box (grey lined) corresponds to a 5-day period and the number of days before measurements are indicated on top of each big box (black lined). Regression coefficients with black color correspond to *p* < 0.05 and white ones to *p* > 0.05.

Leaf burst, expansion and development during spring– as judged by LAI, chlorophylls and LSM–were strongly (R > 0.8), significantly and positively influenced by temperature in all species (Figure 8). Weaker and–in most cases–negative effects appeared for precipitation, with higher precipitation delaying spring leaf development. In contrast to these strong correlations, weaker correlations appear during summer, when the ecophysiological parameters are less fluctuating. Furthermore, the direction of climatic control was reversed during summer in most cases, with higher precipitation and lower temperatures sustaining higher values of LAI, chlorophylls and LSM. During autumn, higher temperatures delayed leaf senescence by positively affecting LAI, chlorophyll content and LSM. The influence of temperature was strong (R > 0.7) and significant. Moreover, the significant negative impact of autumn precipitation in the above-mentioned parameters was related to the advancement of senescence.

As expected, after the winter rainy period, water is ample in the soil and leaf Ψ during spring is high (Figure 6) varying in a small range for all species (−0.36 to −0.90 MPa). Accordingly, spring Ψ data were not further considered for climatic control. However, during the summer period, leaf water potential showed large fluctuations for all species. During that period both temperature and precipitation showed strong correlations with Ψ, with higher rain and lower temperatures sustaining better water status regardless of species (Figure 8). Shorter precipitation intervals compared to summer seem to control Ψ during the autumn period, indicating the recharging capacity of autumn rains. The relationship between temperature and Ψ presented not only the same direction for the three species but also the same time lag of Ψ response, which corresponded to one month during summer and two months during autumn.

Photosynthesis is the most dynamically responsive and most fluctuating parameter among the five studied in this work (Figure 7). Interestingly, it appears there are high correlations with climatic variables during all seasons for all species. Similar to LAI, chlorophylls and LSM, higher temperatures and low precipitation during spring enhance photosynthetic performance. This pattern is reversed during summer, with the relationship with the temperature being significantly negative, though rain events during the dry months favor photosynthesis (*p* > 0.6). However, during autumn both high temperature and rain are necessary to restore photosynthetic depressions of the stressful summer period, with R ranging between 0.68–0.93 in all studied species (Figure 7).

## 4. Discussion

### 4.1. LAI

LAI has been extensively used as a measure of the total quantity of above-ground biomass and subsequently as an estimator of primary productivity, describing how foliage area controls fundamental processes of vegetation-environment interactions, such as water and carbon exchange and light interception [28,29,30]. In the present study, seasonal and inter-annual fluctuations of LAI were assessed in the three studied species for both connecting phenology with plant ecophysiology and identifying the climatic control on this central vegetation parameter. An abrupt increase in LAI during the leaf burst was evident for all species yet was more pronounced in beech. Additionally, the maximum values achieved were higher in beech reaching 9.5 m^2^ m^−2^ comparable with mature Central European forests [28], confirming its late-successional characteristic of forming extensive crown and maintaining high LAI compared to most European hardwood tree species [2]. Mid-summer slight decreases in LAI were apparent every year but were recovered promptly, except for the dry summer of 2007, during which beech showed a continuous decline in LAI, denoting a pre-senescence leaf shedding. This response has been widely observed for beech and is considered an acclimation mechanism in years where a moist spring is followed by a dry summer; the first triggers high leaf number and area whose evaporative demands cannot be supported by the water shortage of the latter [2]. The resulting mismatch is mainly attributed to the deterministic leaf and shoot growth pattern of beech [31], according to which only a few new stems are developed later in the summer, minimizing the tree’s ability to compensate for the above-mentioned foliage loss. We cannot exclude, however, that the apparent mid-summer decline of LAI might have resulted from alterations in leaf inclination and/or leaf curling [29]. *Quercus* species showed a different pattern of LAI decline compared to beech, which started at mid-summer and gradually decreased until the end of the leafy period, in October, without any reversals. A similar partial summer defoliation event has been reported during the extremely dry 2003 for European broadleaf forests [32,33]. Whether it is a drought avoidance mechanism, or a severe consequence of negative climatic water balance is questionable. Nevertheless, its impacts on concurrent and subsequent tree growth are considerable in terms of carbon and nutrient cycles since nutrient resorption cannot take place and the photosynthetic area is lost [3,34]. The latter has been quantitatively demonstrated in oak forests in France, where the premature leaf fall induced by the 2003 drought significantly delayed bud burst and decreased crown vitality of the next year (2004) via reducing the carbohydrate reserves created at the end of the 2003 growing season [33]. Autumn senescence is marked by a steeper decline of LAI in beech compared with the gradual one of oaks, yet the timing does not present variations between years, regardless of species. Autumn senescence is crucial for modulating the duration of the photosynthetically active period and for nutrient resorption for supporting the growth of the following spring [35]. The comparison between the studied species indicates that beech speeds up the nutrients recycling from senescing leaves and starts a concurrent and quick chlorophyll degradation (Figure 4). Leaf senescence is triggered not only by direct climatic effects on leaf status but also by the decrease in the capacity of carbon sinks, which down-regulates photosynthesis, the well-known ecophysiological mechanism of sink limitation [23,24,35]. The cessation of xylem formation and wood growth in both beech and oaks during late summer-early autumn reduces the tree’s sink capacity and happens concurrently with the onset of foliar senescence [24,36].

### 4.2. Chlorophylls and LSM

Chlorophyll content showed a similar seasonal and partly inter-annual fluctuation with LAI in all three studied species. Beech presented an accelerated rate of both chlorophyll content increase and decrease in the leaf-out phase of spring and senescence in autumn, respectively, compared with oak species. Year-to-year differences in chlorophyll maxima during summer were evident in both *Q. cerris* and *Q. frainetto*. Noteworthy is the exceptionally dry 2007, where a mid-August significant decrease was obvious, which was not recovered afterward. Although both oak species experienced the same environmental conditions in the studied mixed forest, they reacted differently in this extreme drought since *Q. cerris* started the chlorophyll decline almost a month later and reached considerably lower values than *Q. frainetto* at the last measurement at the end of October. The chlorophyll loss between summer maximum and minimum values in 2007 exceeded 50%, fairly higher than the 20% reported by Süßel et al. [32] for a mature oak forest in Germany during the extremely hot and dry 2018. Their work confirmed that the vapor pressure deficit, which is affected by temperature, strongly determined the chlorophyll profile, in accordance with the conclusion of Bachofen et al. [34] for beech. The dry 2007 of the present study differentiated the LSM profile of *Q. cerris*, resulting in higher values of this parameter, which otherwise showed the minimum inter-annual and seasonal fluctuations. LSM depicts the investment of biomass per unit area, thus is a good measure of schlerophylly and is very responsive to environmental gradients [37]. Notably, low water availability increases LSM, as was the case with the 2007 drought impacts on *Q. cerris*. Beech did not exhibit noticeable inter-annual variation of LSM, in contrast with the findings of a field survey along a latitudinal gradient in Italy, where the southernmost, thus drier provenance showed higher leaf mass per unit area compared with the other measured populations [38].

### 4.3. Water Potential

During the adverse summer period, a gradual decline of Ψ was evident in all studied species, resulting in a minimum value, the time point of which varied among years. In normal years with regard to precipitation and temperature, the minimum Ψ appeared in September, in contrast with the dry year of 2007, where August was the most unfavorable month. Beech maintained a better water status since its minimum measured Ψ reached −1.7 MPa, yet *Q. frainetto*, with −3.4 MPa and *Q. cerris*, with even lower −3.7 MPa experienced considerable water stress in 2007. Working with beech of similar altitudes in Greece, Fotelli et al. [8] found analogous Ψ drops during August, although in an earlier study of beech response to the exceptionally dry 1998, Raftoyannis and Radoglou [20] reported substantially lower values of even −4 MPa. The beech of the present study did not reach such extremes in the dry 2007; however, the minimum Ψ may be connected with the significant pre-senescence LAI drop during that August, as discussed previously. According to Leuschner [2], the beech bears an extensive fine root system, two-thirds of which is located in the topsoil (0–30 cm), negatively affecting its ability to absorb water due to drought-induced soil desiccation, thus supporting the full crown. Recent reports documented that *Q. cerris* and *Q. frainetto* display an anisohydric behavior characterized by a pronounced decline of mid-day Ψ and a lower stomatal sensitivity under water deficit [22,39] and rely on deep soil water layers. Corroborating our results of minimum Ψ values, Wolkerstorfer et al. [21] reported −3.27 MPa and −3 MPa for *Q. cerris* and *Q. frainetto*, respectively; they consider the combination of low Ψ and the maintenance of hydraulic conductivity as an adaptation mechanism of oaks to water stress. They conclude that both *Quercus* species employ similar mechanisms in coping with seasonal fluctuations in water availability; however, *Q. frainetto* seems to be better adapted to drought.

### 4.4. Photosynthesis

Although access to deep water layers seems adequate for evergreen oaks such as *Q. ilex* to sustain their physiological activity during summer [40], deciduous oaks seem to respond to progressive drought with marked decreases in photosynthetic performance [15,21]. Both oak species in our study exhibited in almost all years a significant drop to a minimum value in their seasonal photosynthetic profile with almost zero values. It was reached in the period between the end of August and mid-September, soon after or at the same time-point of a severe Ψ drop. This drought-induced significant decrease in photosynthesis, paralleled by strong reductions in water potential, was also reported by Wolkerstorfer et al. [21] working with the same species in the Bulgarian mountains. *F. sylvatica* retained high photosynthetic rates until the August minimum, except for the dry 2007, while *Quercus* species already began a gradual decline from July. The response of beech seems to confirm that adult trees operate within a large hydraulic safety margin, sufficient to avoid cavitation [2]. Marginal beech populations, such as the studied one in Italy, were found to have developed evolutionary and/or plasticity-related mechanisms to protect their xylem during water shortages, hence showing high xylem functionality [7], although the loss of hydraulic conductance has been found during the extreme drought of 2018 in Central Europe [34].

### 4.5. Climatic Control on LAI, Chlorophylls and LSM

The assessment of climatic control on the physiological parameters revealed an asymmetric effect of temperature and precipitation on the more stable traits, i.e., LAI, chlorophylls, LSM, compared to the dynamic ones, i.e., Ψ and Aleaf. A pronounced positive effect of spring temperatures was evident in all measured parameters regardless of species. The temperature regime during spring plays more than one role in the initial, increasing phase of the LAI curve in beech and oaks; after the chilling temperatures of the winter required for dormancy release, the spring temperatures fulfill the forcing requirements, which are crucial for budburst [36,41] and have a positive correlation with bud development [42]. In combination with this impact, increasing temperatures in spring, when soil water availability is not limited, greatly favor leaf expansion and the enzymatic processes of pigment biosynthesis, triggering increases in LAI, chlorophyll content, LSM and Aleaf in all three studied species. Precipitation was found to negatively affect both LAI and photosynthetic rates during late spring-early summer, a period of amble water reserves in the soil. A plausible explanation could be the possible connection of increased precipitation in certain years with increased cloudiness and low temperatures, which would contribute to declining photosynthesis and leaf development. In line with this view were the results of Meier et al. [4], who found the same LAI-precipitation relationship in a beech forest in Germany and attributed it to the more important role of temperature in late spring compared to nitrogen supply and soil moisture.

### 4.6. Climatic Control on Ψ and Photosynthesis

On the contrary to the above, precipitation during the preceding two and in *Q. frainetto* three months significantly favors summer Ψ and Aleaf, obviously due to replenishment of soil water and alleviation of possible water stress. Additionally, for all species, these two physiological parameters were negatively influenced by the temperature of several previous intervals, interestingly showing similar time lags of one to two months. We may consider this result a cumulative and indirect deterioration of plant water relations and stomatal opening through the adverse effect of high summer temperatures on soil water status. The large time lag of the response conforms to the well-documented ability of adult trees to store a considerable quantity of water in their branch and trunk xylem, which they withdraw and use during the day to balance their Ψ fluctuations [2]. This solution relieves the short-term water shortage, and thus the negative impact of the high temperature on soil water reserves is presented after a time lag. Autumn temperature controls the timing of leaf senescence, which determines the length of the photosynthetically active period, having large-scale effects on ecosystem carbon balance [23]. Although temperature is not the only factor controlling the onset and duration of the senescence process, it is considered the dominant one for European deciduous forests [23]. High autumn temperatures prolonged the growth period of the three studied species through a positive effect on chlorophyll content and LAI, causing a delay in pigment degradation and the subsequent leaf fall. Autumn temperature has been reported to have a dual role in tree performance, impacting both leaf physiological status and growth. According to Fu et al. [23], sustaining an active photosynthetic machinery for an extended period during autumn, along with the concurrent growth processes taking place at the tree scale (i.e., cell maturation and lignification), results in the maintenance of sink activity, which is considered the mechanism of delaying senescence [35].

### 4.7. Species Differences in Sensitivity to Climate

Species-specific differences in sensitivity to climatic control were minor and mainly related to the temporal range of influence for each environmental factor. Generally, longer preceding periods seem to be effective for *Quercus* compared to beech, notably in spring temperatures. In this case, the between-species differences were obvious in chlorophyll, LSM and photosynthetic rates, possibly reflecting the different growth strategy and subsequently the carbon allocation pattern [10,43]. Radial growth in ring-porous oaks occurs before and during bud burst, thus growth activity starts before leaf expansion, mobilizing stored C to meet the needs of early wood formation [10,17]. We may hypothesize that this earlier growth activity in oaks is subject to the control of temperatures that prevail long before leaf development [44], also influencing the carbon available to support the latter. A different pattern is followed by the diffuse-porous beech, the cambial activity of which starts after budburst and reaches its maximum when leaves are mature [17,19]. Finally, we cannot exclude that the different chilling and forcing temperature requirements that characterize beech and oaks [45] play a direct or indirect role in shaping the periods of temperature influence.

The species’ differences in responses, climate sensitivity and climate memory have important implications for trees’ performance and productivity, especially under the ongoing climate change. Moreover, the relevant information may be useful for designing effective forest management measures. Since the main drawback of all three species concerned water relations and possible drought susceptibility, intensive thinning and harvesting may be inappropriate for both oaks and beech forests of Mediterranean areas. The resulting sparser stands with enhanced radiation interception would result in a warmer microenvironment and increased soil evaporation rate, exacerbating drought and compromising the positive effect of competition release.

## 5. Conclusions

The present study revealed the seasonal and inter-annual fluctuations of important ecophysiological parameters for three dominant species of European deciduous forests and the influence of temperature and precipitation at various time intervals on them. *F. sylvatica* reached higher LAI and photosynthetic rates compared to *Q. cerris* and *Q. frainetto* and retained them for longer periods each year, except for a mid-summer decline during the driest year, 2007. Both oak species exhibited greater seasonal and inter-annual variations of all measured parameters, with the amplitude of change between maximum and minimum values of Aleaf and Ψ being considerable. Climatic control was virtually similar across species in terms of direction and intensity of influence. Spring temperature had a positive role in the increasing phase of all ecophysiological processes while rising autumn temperature showed a beneficial effect in prolonging the photosynthetically active period and retarding senescence. Precipitation showed asymmetric effects on the ecophysiology of beech and oaks, negatively affecting the more stable traits (LAI, Chlorophylls, LSM) during spring and autumn but favoring the dynamic ones, i.e., photosynthesis and Ψ during the adverse summer and the autumn period.

Although there is plenty of information on growth-climate and phenology-climate relationships based on dendrochronological or remote-sensed data, field monitoring of the performance of individual mature trees has been rarely conducted, possibly due to practical difficulties. Nevertheless, it is important to establish a detailed and precise connection between trees’ phenology and physiology and the influence of climate on them in order to understand the multiple drivers and feedback loops of the tree-climate relationship, especially under the ongoing climatic crisis.

## Data Availability

Data available on request from the authors.
